# Segregation mediated heterogeneous structure in a metastable β titanium alloy with a superior combination of strength and ductility

**DOI:** 10.1038/s41598-018-25899-3

**Published:** 2018-05-14

**Authors:** Junheng Gao, John Nutter, Xingguang Liu, Dikai Guan, Yuhe Huang, David Dye, W. Mark Rainforth

**Affiliations:** 1 Department of Materials Science and Engineering, The University of Sheffield, S1 3JD Sheffield, UK; 2 Department of Materials, Imperial College London, SW7 2AZ London, UK

## Abstract

In β titanium alloys, the β stabilizers segregate easily and considerable effort has been devoted to alleviate/eliminate the segregation. In this work, instead of addressing the segregation problems, the segregation was utilized to develop a novel microstructure consisting of a nanometre-grained duplex (α+β) structure and micrometre scale β phase with superior mechanical properties. An as-cast Ti-9Mo-6W alloy exhibited segregation of Mo and W at the tens of micrometre scale. This was subjected to cold rolling and flash annealing at 820 ^o^C for 2 and 5 mins. The solidification segregation of Mo and W leads to a locally different microstructure after cold rolling (i.e., nanostructured β phase in the regions rich in Mo and W and plate-like martensite and β phase in regions relatively poor in Mo and W), which play a decisive role in the formation of the heterogeneous microstructure. Tensile tests showed that this alloy exhibited a superior combination of high yield strength (692 MPa), high tensile strength (1115 MPa), high work hardening rate and large uniform elongation (33.5%). More importantly, the new technique proposed in this work could be potentially applicable to other alloy systems with segregation problems.

## Introduction

Since Ti–13V–11Cr–3Al (mass%) was introduced in the early 1950s^1^, β titanium alloys have been increasingly used in numerous industrial fields, ranging from aerospace, automotive to biomedical devices. They offer high specific strength, good corrosion resistance, a Young’s modulus that is closer to that of human bone and good biocompatibility^2–10^. However, one of the main drawbacks of β titanium alloys is their poor strain hardening behaviour^11,12^, which results in rapid localized deformation (necking) during tensile deformation. Recently, a new family of metastable β titanium alloys have been introduced that exhibit both high ductility and high strain hardening rate which arises from the introduction of transformation-induced plasticity (TRIP) and/or twinning-induced plasticity (TWIP)^11,13–19^. It is well known that an increase of β-stabilizing elements can change the balance of the deformation mechanisms of titanium alloys between martensitic deformation, twinning and dislocation slip^11^. The simultaneous occurrence of TRIP, TWIP and dislocation slip have been observed in Ti-12Mo (hereafter, all compositions are in wt.%)^12^, Ti-9Mo-6W^19^ and Ti-8Cr-1.5Sn systems^15^, which display excellent strain hardening and large uniform elongation.

With the aim of improving low yield strength of TWIP/TRIP titanium alloys, Sun *et al*. reported that low temperature flash aging (150 °C 60 seconds) was effective in increasing the yield strength from 480 MPa to 730 MPa via the nucleation of fully coherent ω phase without excessive modification of the β matrix chemical composition^20^. For these TWIP/TRIP titanium alloys grain refinement would be another effective approach to enhance the yield strength considering the well-known Hall-Petch relationship. Grain size has a strong effect on the twinning stress^21–23^ and the stress to trigger the martensite transformation^24–26^. In TWIP steel^21,27^ the twinning stress increases with decreasing grain size and twin thickness is also influenced by the initial grain size. Gutierrez-Urrutia *et al*.^22^ showed that a Hall-Petch relationship provided a good estimate for the effect of grain size on twinning stress. Martensite transformation during tensile testing of metastable β titanium alloys is associated with a typical double yielding and a strain plateau region (pseudo-elasticity in superelastic alloys) after the first yield in the stress-strain curve^11,12,14,26,28,29^. The influence of grain size on the triggering stress for martensite transformation was reported in Ti-10V-2Fe-3Al^28^ and Ti-16V-3.5Al-3Sn alloys^26^. It is reported^26^ that there are several factors governing the triggering stress for stress-induced martensite formation, such as the chemical free energy, the internal frictional resistance to the movement of the interface between β phase and martensite, the internal elastic energy stored in the matrix due to the formation of martensite and the interfacial energy between β matrix and martensite. Among these factors, both the elastic energy and irreversible energy for work done in overcoming internal friction are resistive terms due to the energy consumed, and both of them are associated with grain size^28^. For a given volume fraction of martensite, the internal frictional resistance increases with a decrease in grain size, while decreasing the grain size reduces the intensity and the size of the region over which the stress field is generated. Therefore, a decrease in grain size can lead to an increase in yield strength as the critical strength for twinning, martensite transformation and dislocation slip are grain size dependent^21–25^. However, there is still no effective approach to refine the grain size of β titanium alloys because of the high tendency towards grain growth when annealing at temperatures above β-*T*_*trans*_^30,31^.

Recently, Xu *et al*. reported that an ultrafine equiaxed duplex (α + β) structure can be achieved in metastable β alloys via high pressure torsion (HPT)/ equal-channel angular pressing (ECAP) +400–600 °C annealing^31–34^. Xu *et al*. reported that, after ECAP processing with an high equivalent strain ~3, the ultrafine structure was confined within shear bands and following aging led to non-uniform α precipitation, resulting in equiaxed and acicular α precipitates inside and outside shear bands, respectively^33^. In order to achieve a complete ultrafine/nanoscale duplex (α + β) structure, Xu *et al*. employed HPT to induce very large strains^31^. As expected, a complete ultrafine duplex (α + β) structure was achieved when subjected to sufficient strain. The formation of this equiaxed ultrafine-duplex structure was attributed to the abundant grain boundaries from the nanocrystalline structure after HPT as nucleation sites and enhanced diffusion attributed to the excess free volume generated during severe plastic deformation, which facilitated the rapid growth of α nucleates to a dimension comparable to the β grains, achieving the ultrafine-duplex (α + β) structure^31–33^. Morevoer, an ultrafine structure was also achieved in a biomedical Ti–35Nb–3Zr–2Ta alloy after ECAP processing^35^.

It is well known that beta-stabilizers such as Mo, Nb, Ta, Cr and Fe segregate easily in β Ti alloys during solidification^36^. The segregation of eutectoid elements such as Fe and Cr results in the formation of inhomogeneous β structures also known as β flecks, which is detrimental to the mechanical performance of the alloy^37^. Many efforts have been made to alleviate/eliminate this segregation^38,39^, for example through extended high temperature solid solution treatment. In the current work, by utilizing the segregation of isomorphous stabilizers (Mo and W) in the solidified state, we propose an energy and time efficient processing technique of cold rolling + low temperature flash annealing of the as-cast alloy to develop a new microstructure consisting of equiaxed nano-grained duplex (α + β) structure and micrometre-scale β grains in a metastable β titanium alloy (Ti-9Mo-6W Wt%).

## Results

### Microstructural analysis

**Figure 1:**
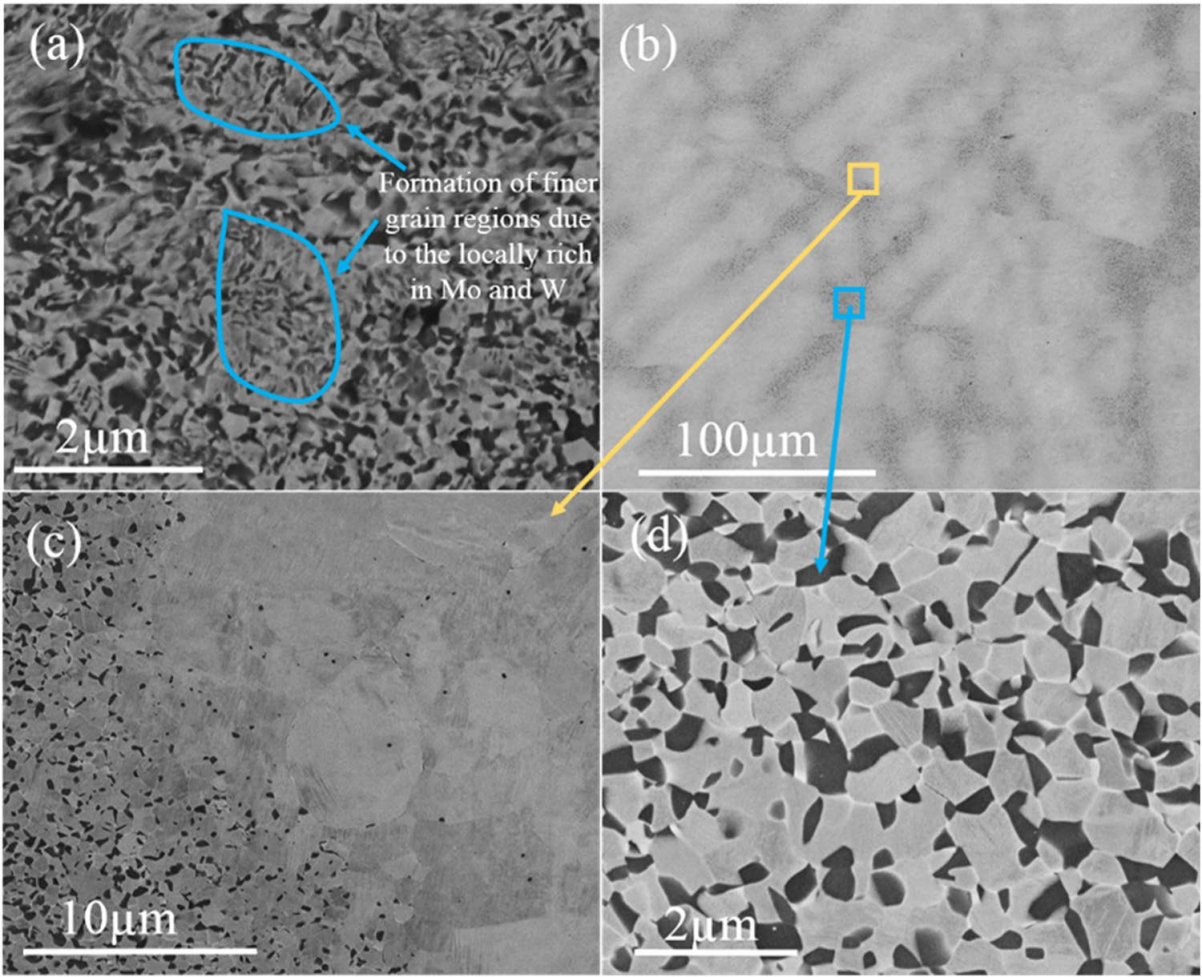
Backscattered SEM micrographs of the Ti-9Mo-6W alloy after annealing: (**a**) 820–2 M and (**b**) 820–5 M. (**c**) and (**d**) are high magnification images of 820–5 M: (**c**) from the interface between black and white regions and (**f**) from the back regions observed in (**b**).

Figure 1 shows the microstructure of the Ti-9Mo-6W alloy after annealing at 820 °C for 2 minutes (820–2 M) and 5 minutes (820–5 M). Figure 1a shows that two kinds of regions with different grain size were observed in the 820–2 M alloy (as marked by the blue circles in Fig. 1a). The relatively larger grain size regions ranged between 40 nm and 540 nm, while the grain size of the regions with finer grains varied between 20 nm and 160 nm. Figure 1b gives a low magnification image for 820–5 M and black-bright contrast regions were observed in Fig. 1b. Figure 1c shows a higher magnification image of the interface between dark and bright regions in Fig. 1b, which indicates that the dark regions in Fig. 1b were composed of equiaxed α (dark) and β (bright) phases, while the bright regions were mainly composed of micrometre β grains (1.0–6.0 µm) and occasional nanometre α grains were also observed at grain boundaries. The higher magnification image (Fig. 1d) from the dark regions further confirmed the coexistence of α (dark) and β (bright) phase in the dark regions. SEM-EDS analysis showed that the average composition in the bright micrometre-grained region was ${{\rm{Ti}}}_{82.4\pm 0.4}\,{{\rm{Mo}}}_{11.1\pm 0.3}{{\rm{W}}}_{6.6\pm 0.3}$ (wt%), while the composition in the nanometre-grained duplex region was in the range ${{\rm{Ti}}}_{88.1\pm 0.8}\,{{\rm{Mo}}}_{8.5\pm 0.5}{{\rm{W}}}_{3.4\pm 0.3}$. The heterogenous distribution of Mo and W was also observed in the as-cast alloy (Sfig. 1, see supplementary materials). According to the Z contrast of back-scattered SEM image, the grain boundaries are poor in Mo and W, while grain interiors are rich in Mo and W. EDS points analysis shows (Stable 1) that the average composition of grain boundaries of as-cast Ti-9Mo-6W alloy is ${{\rm{Ti}}}_{89.2\pm 0.5}\,{{\rm{Mo}}}_{7.9\pm 0.2}{{\rm{W}}}_{2.9\pm 0.3}$, while the average composition of grain interiors is ${{\rm{Ti}}}_{79.5\pm 0.1}\,{{\rm{Mo}}}_{11.3\pm 0.1}{{\rm{W}}}_{9.1\pm 0.1}$.

XRD patterns for all the conditions are shown in Sfig. 2 (see supplementary materials). After cold rolling, the alloy was composed of β phase and martensite α″. For the 820–2 M alloys and 820–5 M, both hcp (α) and bcc (β) peaks were visible and with an increase in annealing time from 2 minutes to 5 minutes, α peaks became weaker, while the β peaks became stronger. Coincident with the loss of the (100), (002), (101), (102), (200) and (201) α peaks after 5 minutes annealing, new peaks corresponding (001) and (002) *ω* peaks appeared.

**Figure 2:**
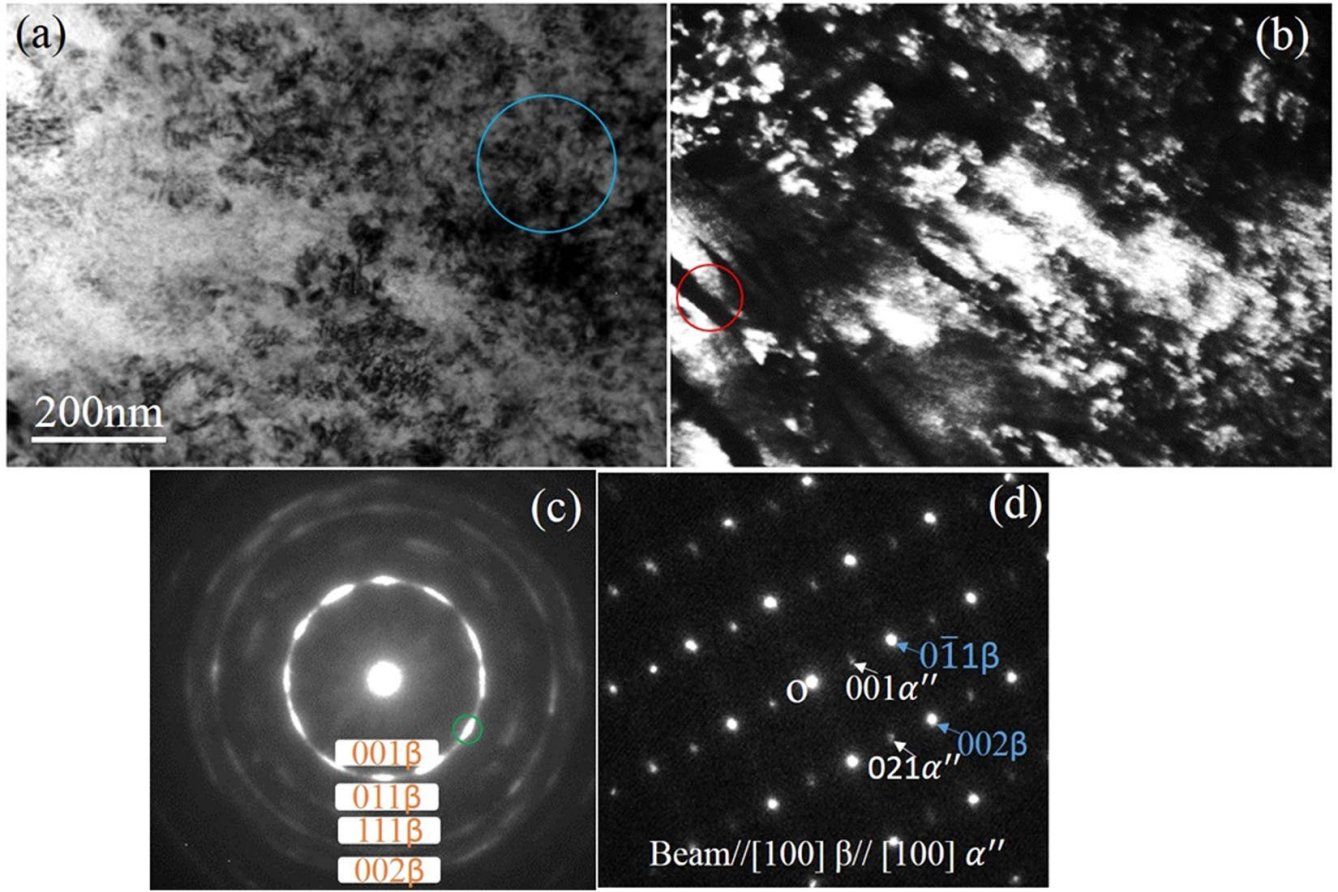
TEM analysis of the cold rolled Ti-9Mo-6W alloy. (**a**) BF-TEM image and (**c**) its indexed SAED pattern recorded from the blue circle marked region in (**a**). (**b**) DF-TEM image recorded using the green circle marked spot in (**c**) and (**d**) its indexed SAED pattern taken from the red circle marked area in (**b**).

TEM analysis was conducted on the cold rolled sample to investigate the microstructural difference between regions rich in Mo and W and regions relatively poor in Mo and W after cold rolling. According to the indexed selected area electron diffraction (SAED) pattern in Fig. 2c, the upper-right corner region in the bright-field TEM (BF-TEM) image (Fig. 2a) comprised nanocrystalline β grains, which was also confirmed by the dark-field TEM (DF-TEM) image in Fig. 2b. The plate-like structure in Fig. 2b was indexed as α″ martensite and β phase, as shown in the indexed SAED pattern in Fig. 2d. The nanocrystalline β grains region was rich in Mo and W and therefore was heavily stabilized and so no martensite transformation occurred during rolling. According to the Mo equivalent criterion^40,41^, the Mo and W content in regions rich in Mo and W can be as high as 11.4 wt.% and 9.2 wt.% (SFigure 1 and Stable 1). The equivalent Mo content in region rich in Mo and W is around 15.4 wt.%, which suppressed martensite transformation during rolling. In contrast, the plate-like structure was relatively poor in Mo and W and was therefore less β stabilized and martensite transformation occurred during rolling.

SFigure 3(a,b) show a BF-TEM image of the 820–2 M and a corresponding SAED pattern (see supplementary materials). Nanoscale grains were clearly observed in sfigure 3a and regions with much finer grains were also observed (marked regions in sfig. 3a), which corresponded well with the SEM observations (Fig. 1a). The indexed diffraction rings in the SAED pattern suggested the presence of both hcp (α) and bcc (β) consistent with the XRD results.

**Figure 3:**
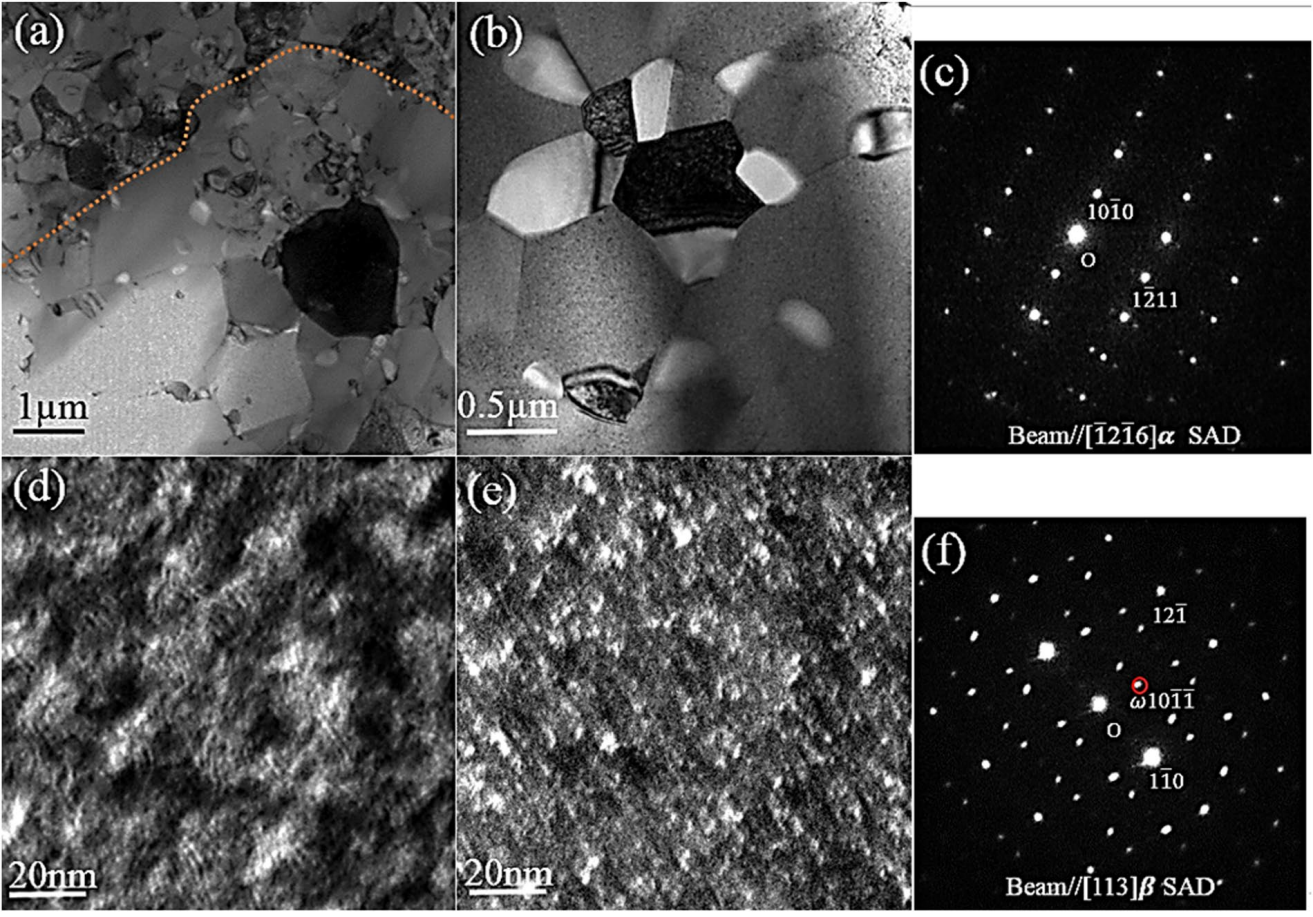
TEM analysis of 820–5 M alloy. (**a**) BF-TEM micrograph taken from the interface between the nanometer-grained region and micrometre-grained region. (**b**) BF-TEM micrograph taken from the nanometer-grained duplex region. (**c**) SAED pattern taken from the grain exhibiting darkest contrast in (**b**). (**d**) High magnification BF-TEM micrograph and its corresponding DF-TEM micrograph (**e**) taken from the bottom-right dark grain in (**a**). (**f**) SAED pattern on [113]β zone axis.

Figure 3a gives a BF-TEM image of the 820–5 M alloy taken from the interface between the nanometre-grained duplex region and micrometre-grained region. The nanometre-grained duplex region shown in the top-left corner of Fig. 3a had a uniform distribution of both α and β phases. In the micrometre-grained region (bottom-right corner), both larger β grains and fine nanoscale grains (40 nm-280 nm) distributed at grain boundaries were observed, which were expected to be α phase. Figure 3b gives a higher magnification BF-TEM micrograph taken from the nanometre-grained duplex region and the $[\bar{1}2\bar{1}6]$ α zone axis diffraction pattern taken from the strongly diffracting grain in Fig. 3b is shown in Fig. 3c. Figure 3d gives a BF-TEM image of the strongly diffracting β grain in the bottom-right corner of Fig. 3a and its [113] β zone axis diffraction pattern is shown in Fig. 3f, which confirmed the presence of *ω* phase in β grains with an orientation relationship [113]β//$[11\bar{2}3]$ *ω*. The corresponding DF-TEM image (Fig. 3e) shows the distribution of *ω* phase, which was recorded from a set of *ω*(10$\bar{1}\bar{1}$) reflections, marked by the circle on the [113]β zone axis SAED pattern in Fig. 3f. The black-white contrast in Fig. 3d was attributed to the spinodal decomposition of the β phase in the water-quenched condition, as observed in Ti-55531^24^. Energy filtered TEM (EF-TEM) analysis confirmed significant heterogeneity in the Mo at the scale of 7–8 nm in the β grain (SFig. 4), consistent with that of Ti-55531 (11 ± 2 nm)^24^.

**Figure 4:**
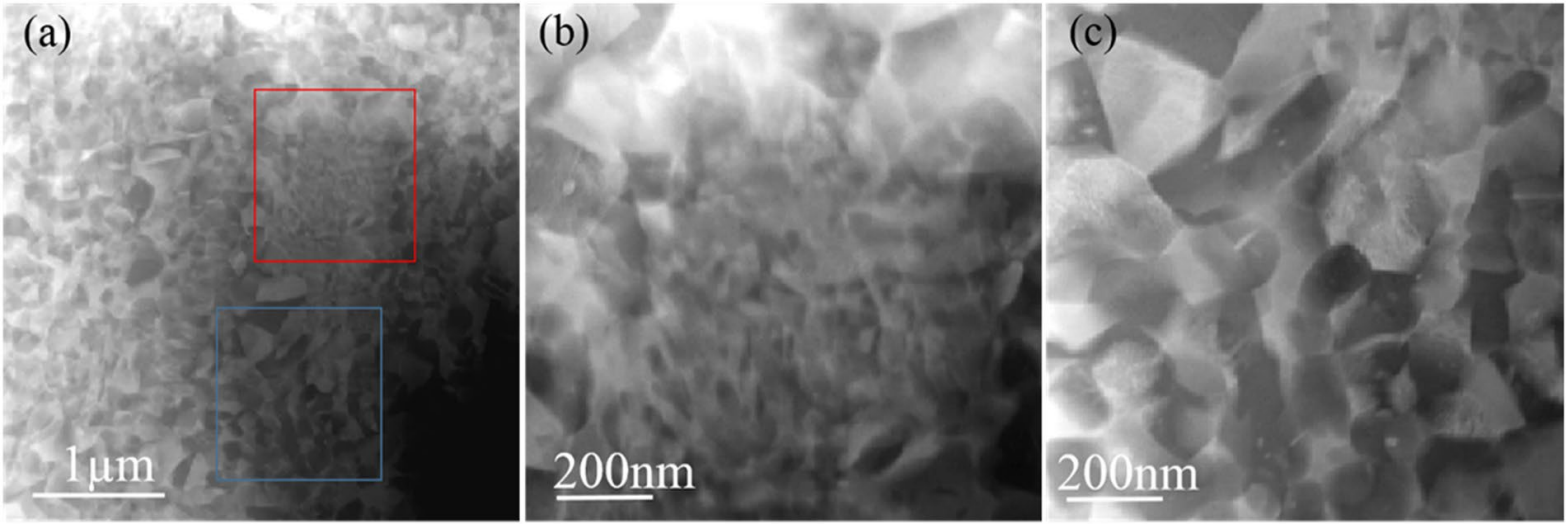
STEM analysis of 820–2 M. (**a**) Low-magnification HAADF image showing heterogeneous nanocrystalline microstructure with relative coarse grain regions and fine grain regions. (**b**) and (**c**) High magnification HAADF images from the red and blue square box marked regions in (**a**), respectively.

STEM was carried out to further investigate microstructural evolution with annealing time and the composition difference between α and β phase in the nanometre sized grains was analysed using EDS in STEM mode with a nominal 1.0 nm electron probe. High-angle annular dark-field (HAADF) (Fig. 4) imaging was found to discriminate the distributions of nanoscale α and β phase through Z contrast, with the higher Mo and W concentration in the β phase giving distinctly brighter contrast and the α giving darker contrast. As shown in Fig. 4a, the nanometre sized duplex (α + β) structure exhibited a relatively homogeneous distribution. Figure 4b shows a high-magnification image from the red square marked finer grain region in Fig. 4a which contains finer α grains with an average grain size 28 ± 9 nm. Figure 4c shows a high-magnification HAADF image from the blue square marked nanometre-grained duplex structure and the average grain size for α and β grains was measured to be 130 ± 68 nm and 146 ± 51 nm, respectively. The volume fractions of both α and β phases in the nanometre-grained duplex structure region were measured to be 55.2% and 44.8%, respectively. Here the volume fraction of α and β phase in the fine grain regions cannot be quantified because of the extremely fine grain size.

**Figure 5:**
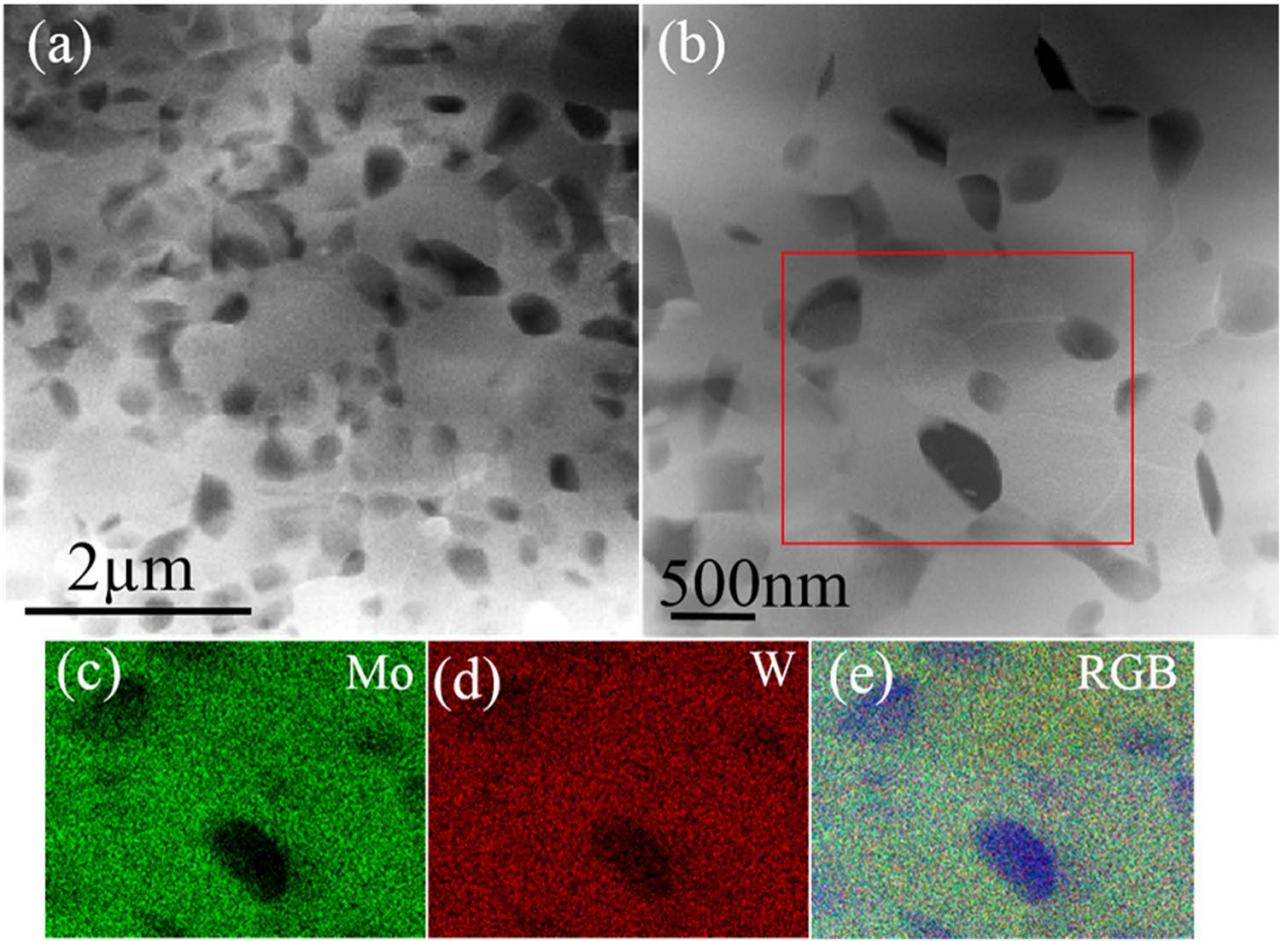
STEM analysis of 820–5 M. (**a**) Low-magnification HAADF image recorded from nanometer-grained duplex structure. (**b**) High-magnification HAADF image from nanometer-grained duplex structure. (**c**),(**d**) and (**e**) Showing the STEM-EDS mapping results of the red square box marked region in (**b**) for Mo, W and RGB map (Mo (green) and W (red)), respectively.

Figure 5a and b give HAADF images from the nanometre-grained duplex structure of 820–5 M. Figure 5a gives a low-magnification HAADF image, which shows the homogeneous distribution of α phase at the grain boundaries of the β phase. For the 820–5 M alloy, a finer grained region was not observed. Moreover, α grains in this duplex structure were much smaller than the β grains, Fig. 5b. The average grain size for the β phase was 599 ± 305 nm, while the average grain size for the α phase was 213 ± 103 nm. The volume fractions of both α and β phases were measured to be 30.8% and 69.2%, respectively. TEM-EDS mapping shown in Fig. 5c–e further confirmed that α phase was depleted in Mo and W, which corresponded well with the contrast in the HAADF images. EDS analysis in STEM mode showed that the average compositions of α and β phases were in the range of ${{\rm{Ti}}}_{83.9\pm 1.1}\,{{\rm{Mo}}}_{9.7\pm 1.9}{{\rm{W}}}_{6.4\pm 0.9}$, respectively.

**Figure 6:**
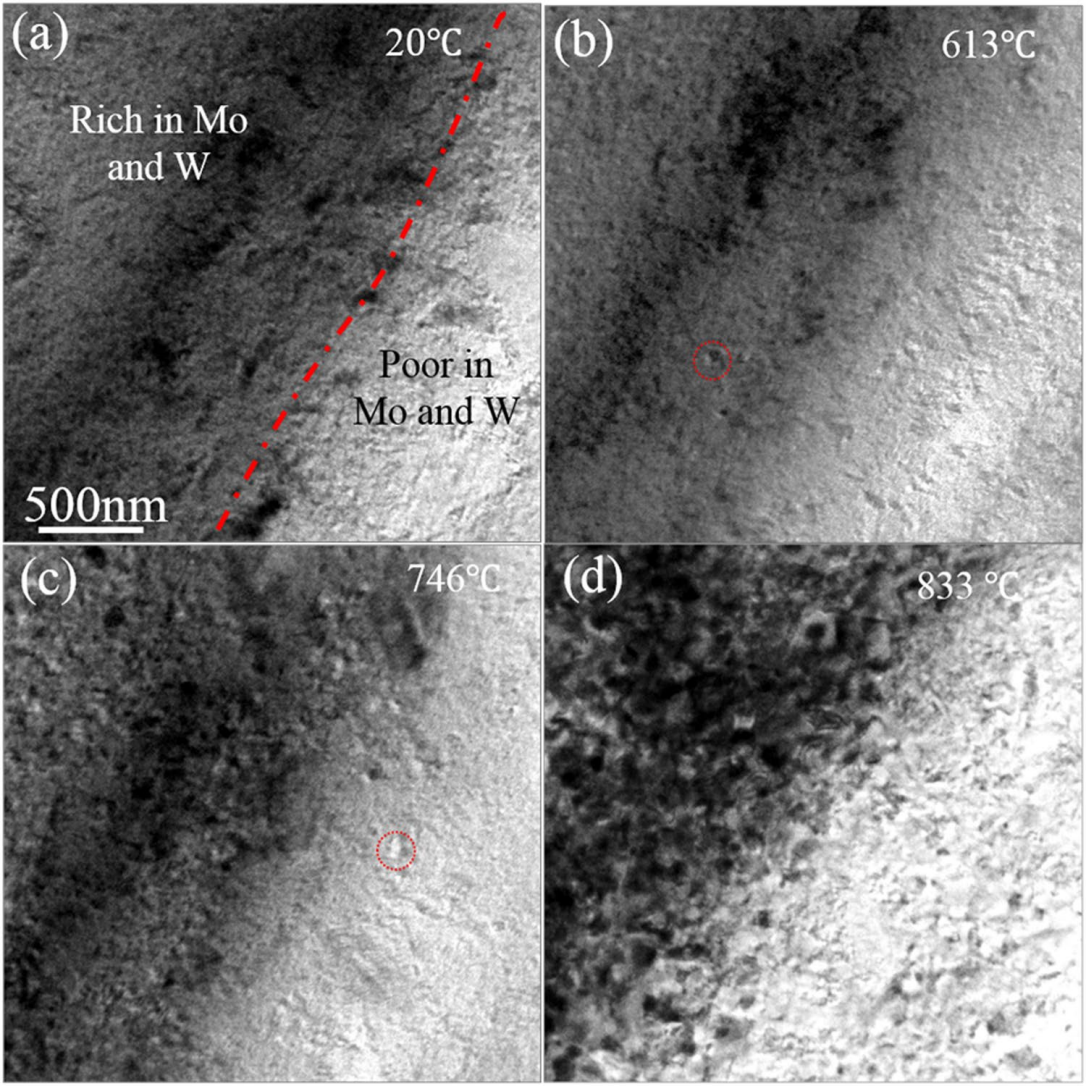
*In-situ* heating TEM-BF images of cold rolled Ti-9Mo-6W alloy: (**a**) as cold-rolled state, (**b**) after heating 154 seconds when reading temperature was up to 613 °C, (**c**) after heating 182 seconds when the specimen holder temperature was up to 746 °C, and (**d**) after heating 201 seconds and the specimen holder temperature was up to 833 °C.

To identify the formation mechanisms of the heterogeneous microstructure, thin foil specimens were heated in the TEM. Figure 6 shows a sequence of images of cold rolled Ti-9Mo-6W during heating in the TEM. The heating curve is shown in Sfig. 5 and two real-time videos are also provided in the supporting materials. Figure 6a shows that there were two kinds of regions due to the heterogeneous distributions of Mo and W, divided by the red dashed line. According to the contrast of the BF-TEM image, the upper-left region was rich in Mo and W, while the bottom-right region was relatively poor in Mo and W. After heating the sample for 154 seconds when the sample holder temperature reached 613 °C, the first recrystallized grain was observed in the Mo and W rich regions, as marked by the red circle in Fig. 6b, while for the region relatively poor in Mo and W, no recrystallization was observed. It should be noted that the temperature of the hot stage control unit was not the same as the specimen temperature (due to thermal losses from the specimen), which was estimated to be about 100 °C lower than the actual temperature of sample^42^. When the temperature was increased to 746 °C for 154 seconds (Fig. 6c), the first recrystallized grain was observed in the region relatively deficient in Mo and W, while for the region rich in Mo and W, extensive recrystallization occurred. When the temperature was increased up to 833 °C for 201 seconds (Fig. 6d), a fully recrystallized microstructure was observed in the Mo and W rich region. At this stage, recrystallization was still continuing in the region deficient in Mo and W. See dynamic details from supplementary videos.

### Mechanical properties

**Figure 7:**
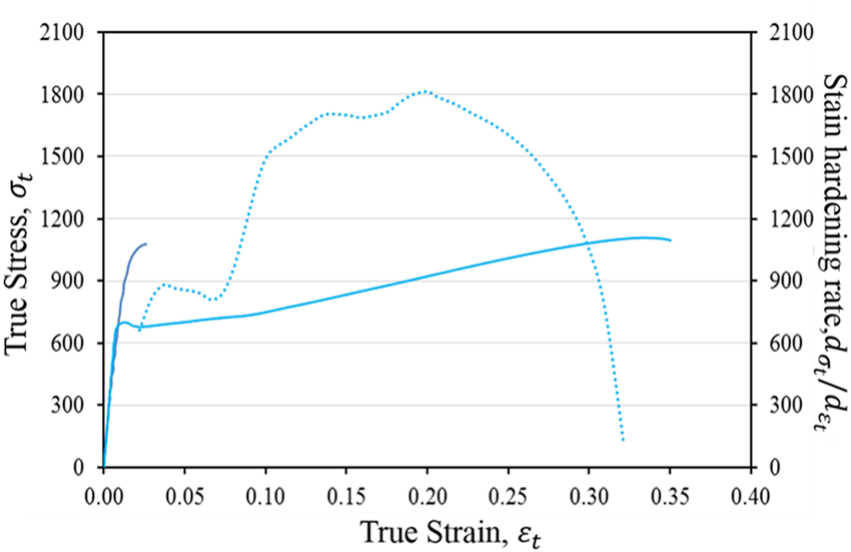
(**a**) Room-temperature true stress-strain curves of 820–2 M and 820–5 M alloys at a strain rate of 5 × 10^−4^ in tension. The corresponding strain hardening rate as a function of true strain for 820–5 M was shown in dashed line.

The true tensile stress-strain curves for 820–2 M and 820–5 M are shown in Fig. 7. The 820–5 M alloy yielded at 692 MPa, after which minor strain hardening occurred to a peak stress of 701 MPa at a strain of 1.2%, followed by strain softening with a lowest stress of 678 MPa at a strain of 2.1%. After strain softening, pronounced strain hardening occurred with an initially monotonic increase in stain hardening rate was observed, leading to an ultimate tensile strength of 1115 MPa with a uniform strain of 33.5%. For the 820–2 M alloy, yielding occurred at 940 MPa, followed by strain hardening to an ultimate tensile stress of 1086 MPa at a uniform strain of 2.6%. The corresponding strain-hardening rate (d*σ*/d*ε*) (dashed line) of the 820–5 M alloy exhibited a multi-stage deformation process. After yielding, the strain hardening rate dropped rapidly, followed by a rapid increase to 887 MPa at a strain of 0.037. From 0.037 to 0.072, the strain hardening rate stabilized around 880 MPa. Beyond a strain of 0.072, the strain hardening rate rose again and reached 1718 MPa at a strain of 0.14. The strain hardening rate fluctuated around 1700 MPa and the peak value was 1816 MPa at a strain of 0.20. After that, the strain hardening rate starts decreasing steadily.

### TEM analysis of the tensile tested alloys

**Figure 8:**
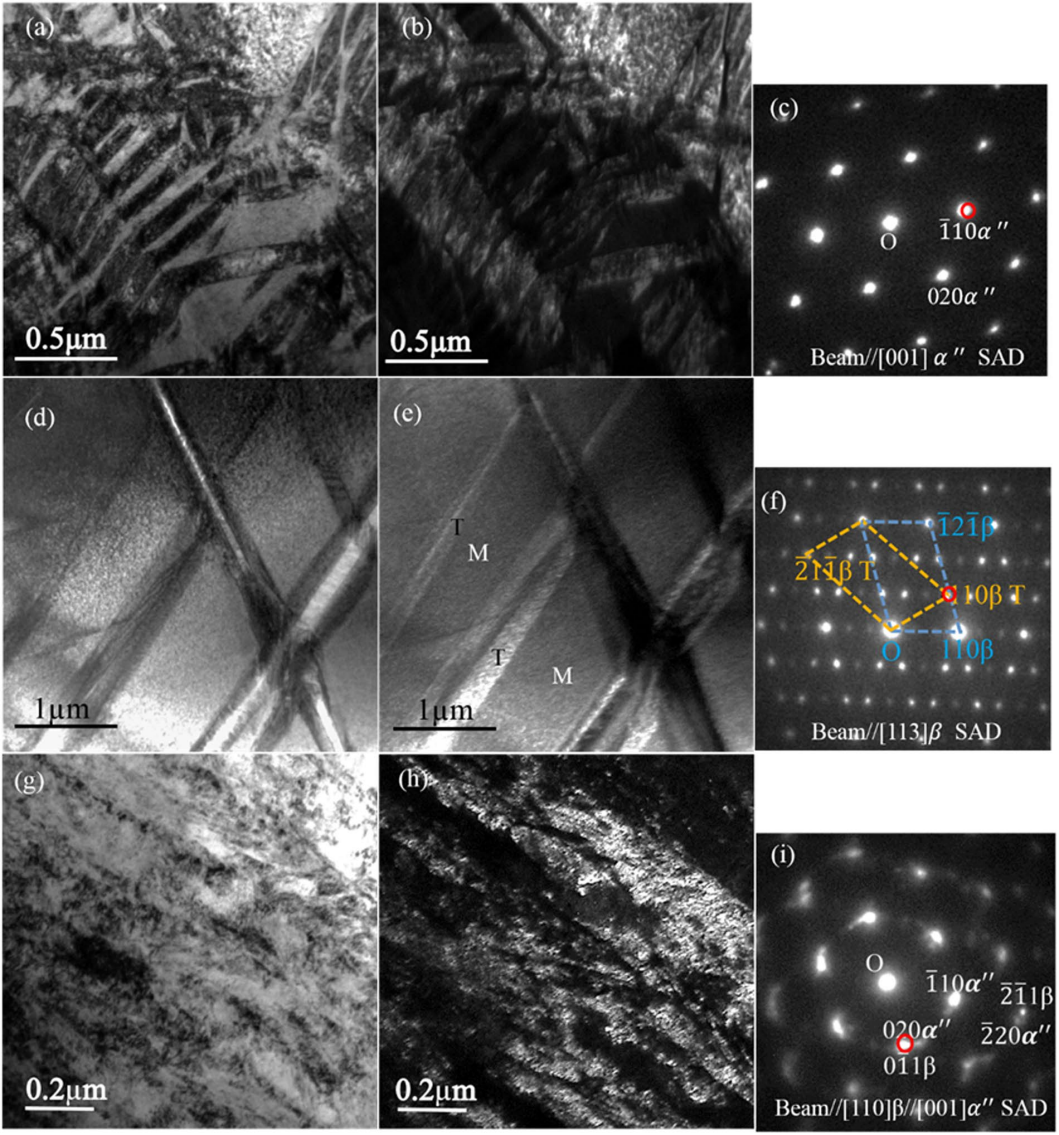
TEM analysis of the 820–5 M alloy deformed to a strain of 2.1% and 38% (after necking), respectively. (**a**), (**b**), (**c**), (**d**), (**e**) and (**f**) for the specimen deformed to a strain of 2.1%. (**a**) BF-TEM image recorded from ultrafine β grain. (**b**) Corresponding DF-TEM image by selecting *α*″ ($\bar{1}10$) diffraction spot (highlighted by red circle in (**c**)). (**c**) *α*″ [001] zone axis SAED pattern recorded from the plate-like structure in (**a**). (**d**) BF-TEM image taken from the micrometre-grained β phase. (**e**) Corresponding DF-TEM image recorded from a set of β(110) twinning reflections (red circle in (**f**)). (**f**) β[113] zone axis SAED pattern. (**g**), (**h**) and (**i**) for the specimen deformed to a strain of 38%: (**g**) BF-TEM image. (**h**) Corresponding DF-TEM image recorded by selecting *α*″ (020), highlighted by the red circle in (**i**). (**i**) SAED pattern.

In order to understand the deformation mechanism of the 820–5 M alloy, TEM specimens were removed from tensile samples tested to a strain of 2.1% and 38%. Figure 8a presents the BF-TEM image taken from a nanometre β grain region after tensile testing to a strain of 2.1%. As shown in Fig. 8a, plate-like *α*″ martensite was formed in the nanometre β grains, with a width in the range of 60nm–265nm. No twinning was observed in the nanometre β grains. The BF-TEM image shown in Fig. 8d was recorded from a micrometre β grain, and the indexed SAED pattern shown in Fig. 8f identified {112}〈111〉 β twins. The DF-TEM image in Fig. 8e was taken using a β(110) twin diffraction spot. The BF-TEM image (Fig. 8g) and its corresponding DF-TEM image (Fig. 8h) were taken from the deformed microstructure with a tensile strain of 38%. Figure 8h showed the plate-like martensite (*α*″) structure. The indexed SAED pattern in Fig. 8i showed that the deformed microstructure consisted of *α*″ and β phases with an orientation relationship [110]β//[001] *α*″. The DF-TEM image shown in Fig. 8h shows much finer *α*″plates with a width in the range of 20–82 nm near the primary *α*″plates.

## Discussion

In the current work, based on the pioneering work of Xu *et al*. who engineered a complete duplex structure in metastable β titanium alloy^31–33^, we developed a heterogeneous structure consisting of nanometre-grained duplex (α + β) structure and micrometre scale β grains by utilizing solidification segregation of β stabilizers. The heterogeneous distribution of Mo and W after solidification resulted in a variation in the structure after cold rolling. The regions rich in Mo and W exhibited nanocrystalline structure, while for the region deficient in Mo and W, plate-like martensite laths were observed. The β phase in regions rich in Mo and W was heavily stabilized, so the high strain induced by cold rolling was accommodated by extensive dislocation activity, such as dislocation multiplication, accumulation, interaction, tangling, and spatial rearrangement. The dislocation cell structure subdivided a grain into many fine grains at the nanometre scale^13,31,32,43–45^. In contrast, in the β phase regions containing fewer β stabilising elements, the deformation strain was accommodated by martensite transformation^13,32,43,44,46^. These segregation mediated structures exhibited different recrystallization behaviour. The abundant dislocation and subgrain boundaries in the nanocrystalline regions resulted in a higher driving force for recrystallization. Moreover, these defects can act as preferential nucleation sites for α precipitation and enhanced atomic diffusivity can accelerate α nucleation and growth^31,33,34^. The heating experiments showed that nucleation of precipitates started from the heavily stabilized nanocrystalline region when the sample holder temperature reached 613 °C (Fig. 6b). Although is hard to tell whether the precipitated grains were α or β just from TEM observation, it was expected that they were α grains given that the sample temperature was estimated to be roughly 500 °C, which is in the range generally used as an aging temperature (450 °C to 600 °C) for α precipitation in metastable β titanium alloys. Similarly, Zheng *et al*. reported that during isothermal aging at 350 °C for 90 mins refined α precipitates formed in Ti5553 alloy^47^. Moreover, α precipitation also occurred in a HPT-processed Ti-5553 alloy during a short ageing at 600 °C for 30 s^34^.

The first recrystallized grain in the less stabilized region was observed when the sample holder temperature reached 746 °C (sample temperature ~646 °C) (Fig. 6c). At this temperature significant recrystallization and α precipitation occurred at the heavily stabilized nanocrystalline region, which is a temperature close to the estimated recrystallization temperature (~700 °C) of cold rolled VT-22 alloy^48^. Simultaneous occurrence of β recrystallization and α precipitation is possible, as proposed in the HPT processed Ti5553 alloy^34^. The increase of sample holder temperature to 833 °C (sample temperature ~733 °C) led to extensive β phase recrystallization and α precipitation in the whole material (Fig. 6d). A further increase in temperature resulted in loss of the edge of electron transparent regions in the sample (see video in the supporting materials). Regrettably, further microstructural evolution could not be observed by *in-situ* TEM as the sample degraded. However, as noted before, the STEM results shown in Figs 4 and 5, taken after the formation of uniform duplex structure, a further increase in temperature and time resulted in α + β to β transformation in the region rich in Mo and W, while for the less stabilized regions, both the α + β to β and its reverse transformation occurred, although α + β to β tranformation was more thermodynamically favourable. The observation of finer duplex regions in the 820–2 M alloy (Fig. 4a) in the region rich in Mo and W was a result of the α + β to β transformation in the heavily stabilized regions. In contrast, in the less stabilized region, although the volume fraction of α decreased from 55.2% to 30.8% with an increase in annealing time from 2 to 5 minutes (Figs 4 and 5), the average grain size of α phase increased, which confirmed α + β to β transformation dominated the process and its reverse transformation occurred as well.

The triggering stress for stress-induced martensite transformation in metastable β titanium alloys is dependent on the following two independent factors: (i) the chemical stability of the β phase and (ii) the β domain size (in the current work this is defined as the β grain size)^16,25,26^. Both the increase of β phase stability and the decrease of β grain size oppose the stress-induced *α*″ martensitic transformation because chemically stable β phase tends to hinder β to *α*″transformation and the accommodation of the shape change introduced during β to *α*″ transformation is more difficult with decreasing β domain size^13,49–51^. Moreover, the decrease of the parent β grain size reduces the number of potential nucleation sites for martensite and also restricts the interfacial energy of martensite, resulting in a decrease of martensite start temperature, M_s_^52^. The decrease of M_s_ increases the critical stress and mechanical work for martensite transformation^16^. In the current work, the high yield strength of the 820–5 M alloy (692 MPa) can be attributed to its small grain size and relatively high content of β stabilizers, which result in an increase of critical stress for dislocation slip and martensite transformation. The formation of α phase in the nanometre-grained duplex region led to β phase enriched in β stabilizers; both the nanometre β grains $({{\rm{Ti}}}_{83.9\pm 1.1}\,{{\rm{Mo}}}_{9.7\pm 1.9}{{\rm{W}}}_{6.4\pm 0.9})$ and micrometre β grains $({{\rm{Ti}}}_{82.4\pm 0.4}\,{{\rm{Mo}}}_{11.1\pm 0.3}{{\rm{W}}}_{6.6\pm 0.3})$ contain larger Mo and W contents than the nominal composition (Ti-9Mo6W). The M_s_ was calculated using the average composition of nanometre β grain ${{\rm{Ti}}}_{83.9}{\text{Mo}}_{9.7}{{\rm{W}}}_{6.4}$ and micrometre β grains ${{\rm{Ti}}}_{82.4}{\text{Mo}}_{11.1}{{\rm{W}}}_{6.6}\,$without considering the effect of grain size, according to^51^:

$${{\rm{M}}}_{{\rm{s}}}=1156 \mbox{-} 150{{\rm{Fe}}}_{{\rm{wt}}. \% }-96{{\rm{Cr}}}_{{\rm{wt}}. \% }-49{{\rm{Mo}}}_{{\rm{wt}}. \% }-37{{\rm{V}}}_{{\rm{wt}}. \% }-17{{\rm{Nb}}}_{{\rm{wt}}. \% }-7{{\rm{Zr}}}_{{\rm{wt}}. \% }+{{\rm{Al}}}_{{\rm{wt}}. \% }$$

The M_s_ for the nanometre β grains, micrometre β grains and the nominal composition (Ti-9Mo6W) was calculated to be 270 °C, 197 °C and 313 °C respectively, with the effect of the W being calculated using the Mo equivalent criterion given by Refs^40,41^. Therefore, in comparison with the nominal composition, the higher yield strength of 820–5 M is partially attributed to the much lower M_s_ of both nanometre β grain and micrometre β grains, because the lower M_s_ requires greater critical strength to initiate the martensite transformation. Athough the M_s_ for the nanometre β grains and micrometre β grains are much higher than room temperature, no martensite was observed in the annealed alloy (Figs 1, 3, 4 and 5). This could be attributed to the fine grain size and nanoscale heterogenous distribution of Mo and W (Sfig. 4) that elastically confined martensite transformation, as observed in Ti2448 alloy^53,54^. The suppression of martensite transformation due to the nanometre heterogeneous distribution of Mo and W also leads to an decrease of M_s_, resulting a higher yield strength. In addition, the formation of *ω* in β grains also contributed to the high yield strength. As suggested by Sun *et al*.^20^, the althermal *ω* precipitate/matrix interface maintains a high degree of coherency, which results in elastic strain fields and consequent hardening of the surrounding β matrix, leading to an increase of yield strength.

After yielding, a softening stage was observed for the 820–5 M alloy, which was attributed to stress-induced martensite transformation and the suppression of mechanical twining in the nanometre β grains^55,56^. As observed in Fig. 8a, the specimen with a strain of 2.1% (after softening) showed a large amount of *α*″ martensite in the nanometre β grains, while no twins were observe in the β phase. Although $\{112\}111$ twinning was occasionally observed in micrometre β grains (Fig. 8d,e), stress-induced martensite transformation dominated the initial stage of plastic deformation, leading to the observed softening. The formation of {112}〈111〉 twins, instead of {332}〈113〉 twins, in the micrometre β grains was attributed to the increase of β stabilizers in micrometre β grains^13,57,58^. With further straining, both mechanical twinning and martensitic transformation started to form in the micrometre β grains, and further β to *α*″ transformation required a higher stress in the partially consumed β grains^17^ and combined with a size confining effect for the nucleation and growth of deformation products such secondary *α*″ phase and secondary twinning(Fig. 8h), which led to the moderate strain hardening rate and an abrupt increase of strain hardening rate beyond 7.4%. The formation of finer *α*″ plates could be attributed to the confinement effect of {112}〈111〉 twins or primary *α*″ plates formed in the early stage of plastic deformation (Fig. 8a,d). The formation of geometrically necessary dislocations is a result of the heterogeneous microstructure, with a large grain size difference and the presence of two phases which also contributed to the exceptionally high work hardening rate^59,60^. Sfigure 6 shows that the nanometre equiaxed α grains were significantly elongated towards the loading direction, indicating that the nanometre equiaxed α grains participated in the plastic deformation during tensile testing. It is worth noting that, in comparison with 820–2 M with a complete nano-grained duplex structure, 820–5 M exhibited a better combination of strength and ductility. This suggests that by changing the relative volume fraction of nano-grained duplex structure and micometre β grains as a result of changes in the annealing temperature and time, a series of materials can be developed with different balance of strength and ductility to meet different engineering application requirements.

## Methods

The ternary Ti–9Mo–6W alloy was produced by arc melting in a water-cooled copper crucible using high purity metals (>99.7%) under the protection of high purity Ar. The alloys were melted at least 10 times in total and were flipped between each melting stage. Casting was undertaken in a water cooled copper mould with a slot of 6 mm × 7mm and 30 mm in length. The as-cast materials were cold rolled without homogenization from 6 mm to 1.5 mm in thickness. The cold rolled sheets were annealed at 820 °C for 2 and 5 minutes and quenched in water. The annealing temperature of 820 °C was determined by a series of experiments to give an annealing temperature above the β-trans for regions rich in Mo and W and below the β-trans for regions relatively poor in Mo and W. The annealed samples are designated according to temperature and time, namely, 820–2 M and 820–5 M. Tensile samples with a gauge dimension of 3 mm × 12.5 mm × 1.5 mm were cut and polished from the plate after heat treatment. Tensile tests were performed on a Zwick/Roell Z050 with laser extensometer at a strain rate of 4.0 × 10^−4^ s^−1^ At least 3 samples were tested for each condition. X-ray diffraction was performed on a Siemens D5000 diffractometer fitted with a CuKα radiation source, at a scan rate of 0.1°/min and a step size of 0.01°. The microstructure after heat treatment was analysed by scanning electron microscopy (SEM; FEI Inspect F FEG SEM) with an energy-dispersive spectrometer. After grinding and polishing, SEM samples were polished in Gatan broad ion polishing system (PECS) at 5 kV, 1RPM, 4° for 1 h and then 1 kV, 3RPM, 2° for 0.5 h. Transmission electron microscopy (TEM) was undertaken on a FEI Tecnai 20 operating at 200 kV to analyse both the recrystallized microstructure and the microstructure after tensile testing. Scanning transmission electron microscopy (STEM) was conducted on a JEOL 2010F equipped with an energy dispersive X-ray spectrometer (EDX; Oxford Instruments) and a Gatan Imaging Filter. The chemical composition was obtained in STEM mode with a nominal 1.0 nm electron probe. In order to investigate the effect of segregation on the recrystallization process, a JEOL 3010 microscope was used to record the microstructure evolution during heating of the cold rolled samples by using a GATAN model 628 heating holder. The heating rate was 5 K/s, see the heating and cooling curve in Sfig. 5. TEM specimens were prepared by mechanical grinding followed by ion milling using a Gatan Precision Ion Polishing (PIPSII) system.

### Data Availability

The datasets generated during and/or analysed during the current study are available from the corresponding author on reasonable request.

## Electronic supplementary material

Supplementary materials

In-situ TEM heating of Ti-9Mo6W alloy after cold rolling

In-situ TEM heating of Ti-9Mo6W alloy after cold rolling
