# Supplementary material for: Segregation mediated heterogeneous structure in a metastable β titanium alloy with a superior combination of strength and ductility
Source: Sci Rep. 2018 May 14;8:7512. doi: 10.1038/s41598-018-25899-3 (PMC5951864; doi:10.1038/s41598-018-25899-3)
Supplement: Supplementary file 1 — Supplementary materials [file 41598_2018_25899_MOESM1_ESM.docx]

Segregation mediated heterogeneous structure in a metastable β titanium alloy with a superior combination of strength and ductility

Junheng Gao ^a^, John Nutter ^a^, Xingguang Liu ^a^, Dikai Guan ^a^, Yuhe Huang ^a^, David Dye ^b^, W. Mark Rainforth^a,^*

^a Department of Materials Science and Engineering, The University of Sheffield, S1 3JD, UK^

^b Department of Materials, Imperial College London, SW7 2AZ, UK^


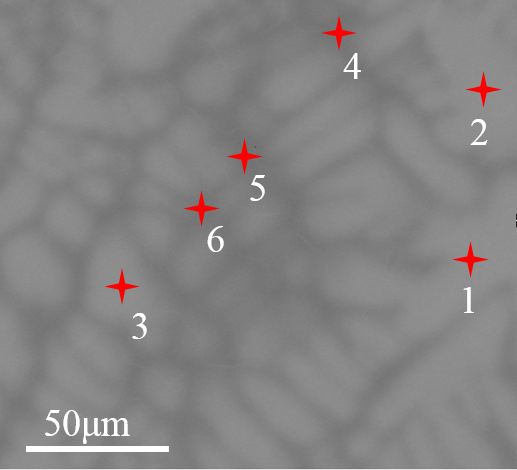


Sfigure 1. Backscattered SEM micrograph of as-cast Ti-9Mo-6W alloy. The 6 red stars marked regions are the EDS point analysis areas. The EDS results are shown in Stable 1.


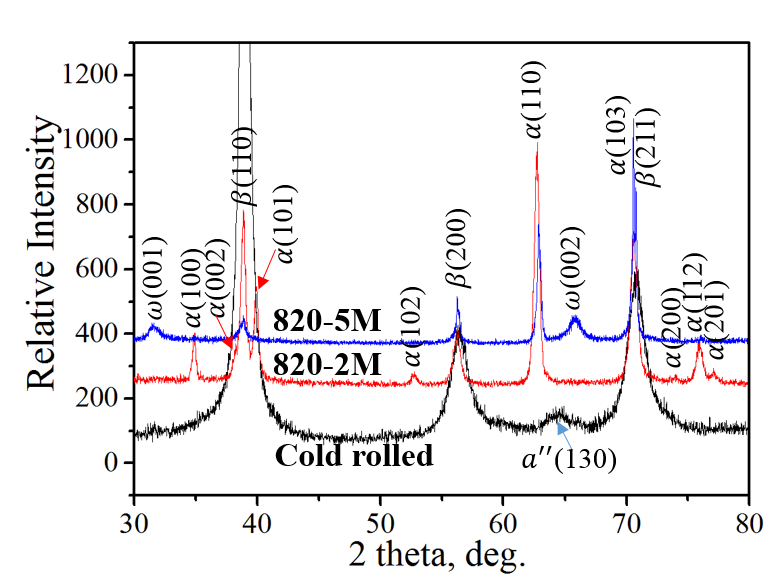


Sfigure 2. XRD patterns of cold rolled, 820-2M and 820-5M alloys, respectively.


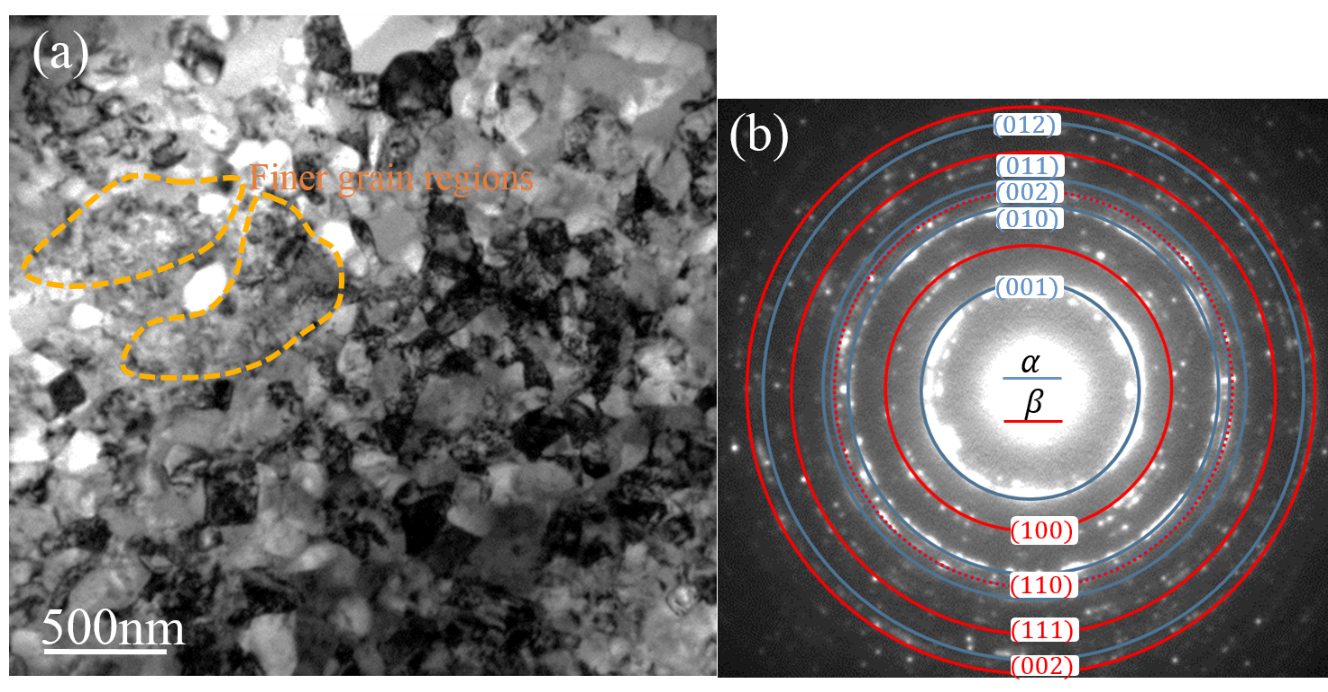

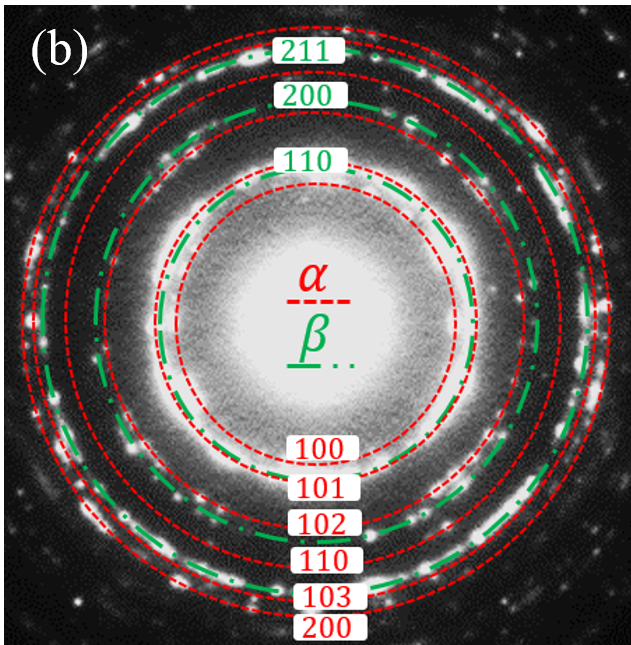


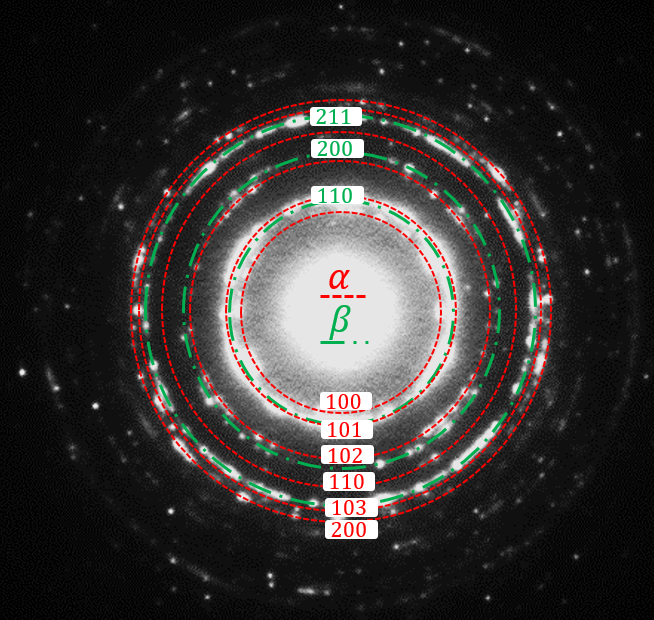


(b)

Sfigure 3. TEM analysis of 820-2M alloy. (a) BF-TEM image of 820-2M and (b) indexed SAED pattern.


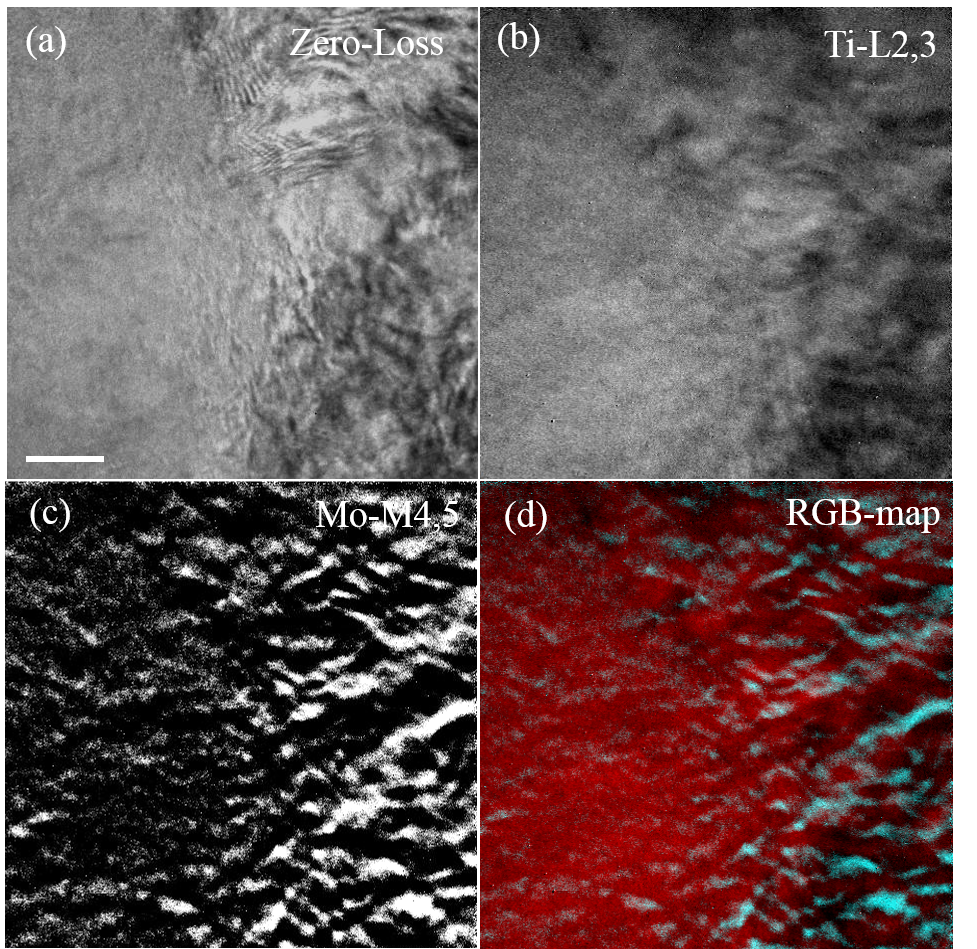


20nm

α Phase

$\beta$ Phase

Sfigure 4. EF-TEM analysis of 820-5M, showing inhomogeneous distribution of Mo between α and $\beta$ grains and within the $\beta$ grains. (a) Zero-loss micrograph taken from the grain boundary between α and $\beta$ grains. (b) Ti-L_2,3_ map, showing heterogeneity in the Ti composition in the $\beta$ grains. (c) Mo-M_4,5_ map, the brighter contrast in$\beta$ grain indicates regions rich in Mo. A heterogeneous distribution of Mo was observed, particularly in the right hand $\beta$ grain. (d) RGB map obtained using Ti (red) and Mo (blue) maps.


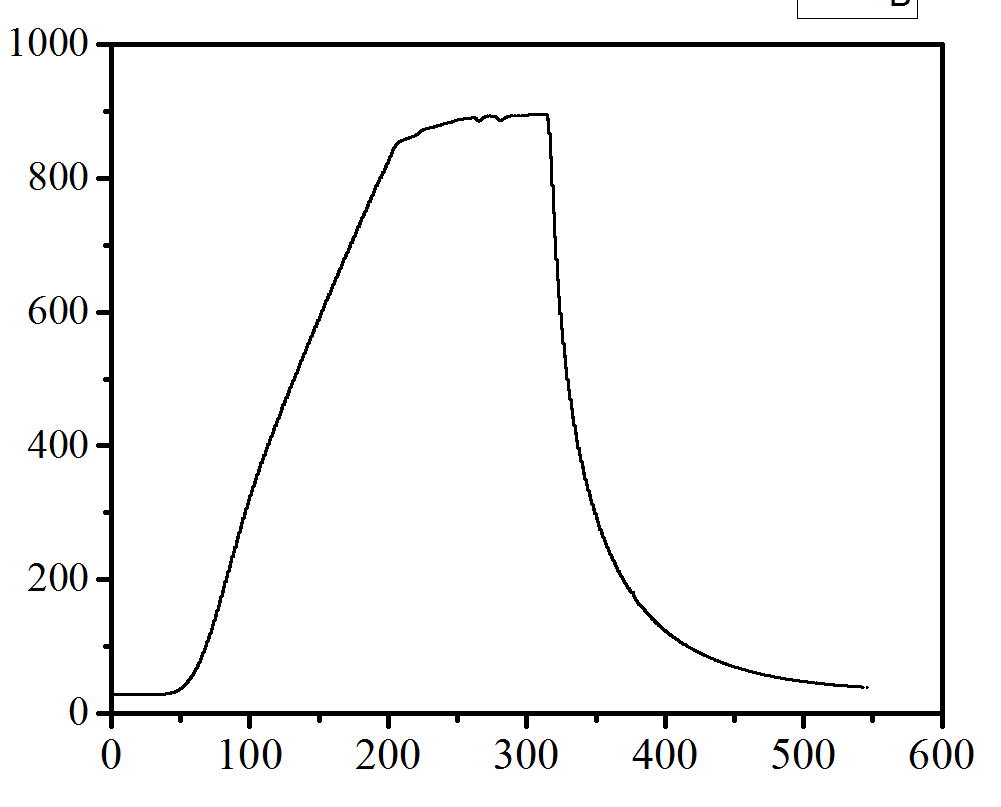


Heating time (S)

Temperature ($℃$)

Sfigure 5. In-situ TEM experiment heating and cooling curve of as-rolled alloy.


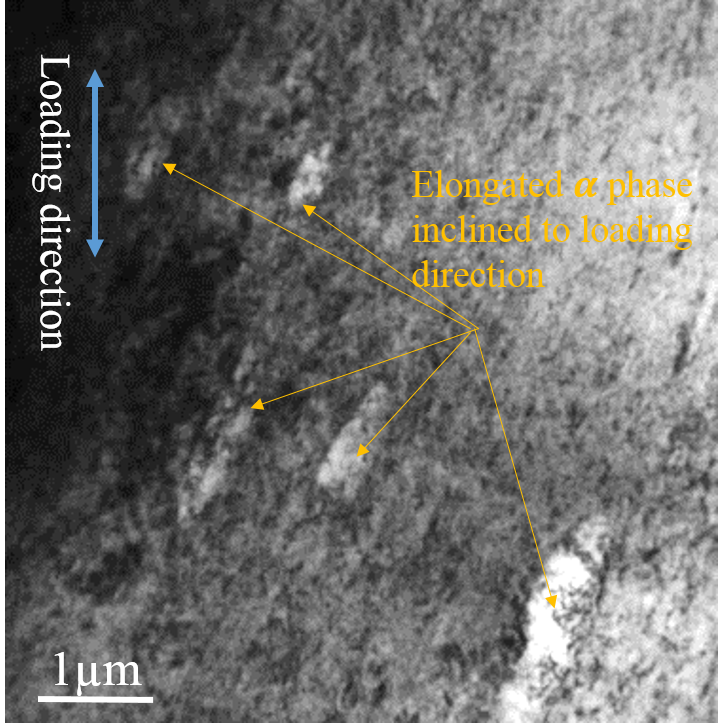


Sfigure 6. TEM analysis of 820-5M alloy deformed to a strain of 38% (after necking). Greatly elongated 𝜶 phase inclined to the loading direction was observed.

Stable 1. Chemical compositions of grain boundaries and grain interiors for as-cast Ti-9Mo-6W alloy.

|  | **Ti (wt.%)** | **Mo(wt.%)** | **W(wt.%)** |
| --- | --- | --- | --- |
| Site 1 | 79.7 | 11.3 | 9.0 |
| Site 2 | 79.4 | 11.4 | 9.2 |
| Site 3 | 79.5 | 11.3 | 9.2 |
| Site 4 | 89.2 | 7.9 | 2.9 |
| Site 5 | 88.7 | 8.1 | 3.2 |
| Site 6 | 89.8 | 7.7 | 2.5 |
